# Co-Occurring Post-Traumatic Stress Disorder and Alcohol Use Disorder in U.S. Military and Veteran Populations

**DOI:** 10.35946/arcr.v39.2.06

**Published:** 2018

**Authors:** Emily R. Dworkin, Hannah E. Bergman, Thomas O. Walton, Denise D. Walker, Debra L. Kaysen

**Affiliations:** Emily R. Dworkin, Ph.D., is a postdoctoral fellow in the Department of Psychiatry and Behavioral Sciences, University of Washington, Seattle, Washington. Hannah E. Bergman, Ph.D., is a postdoctoral fellow in the School of Social Work, University of Washington, Seattle, Washington. Thomas O. Walton, M.S.W., is a graduate student in the School of Social Work, University of Washington, Seattle, Washington. Denise D. Walker, Ph.D., is an associate professor in the School of Social Work, University of Washington, Seattle, Washington. Debra L. Kaysen, Ph.D., is a professor in the Department of Psychiatry and Behavioral Sciences, University of Washington, Seattle, Washington

**Keywords:** addiction, alcohol use disorder, post-traumatic stress disorder, military, veteran

## Abstract

Co-occurring post-traumatic stress disorder (PTSD) and alcohol use disorder (AUD) are costly and consequential public health problems that negatively affect the health and well-being of U.S. military service members and veterans. The disproportionate burden of comorbid PTSD and AUD among U.S. military service members and veterans may be due to unique factors associated with military service, such as aspects of military culture, deployment, and trauma exposure. This review addresses the prevalence of co-occurring PTSD and AUD in military and veteran populations, population-specific factors that contribute to development of the comorbid conditions, and evidence-based treatments that have promise for addressing these conditions in military and veteran populations. Future directions for research and practice relevant to military and veteran populations are discussed.

Post-traumatic stress disorder (PTSD) and alcohol use disorder (AUD) are costly and consequential public health concerns that have disproportionately affected U.S. military service members and veterans.^1^,^2^ Understanding the co-occurrence of PTSD and AUD is especially important because of the negative implications for the health and well-being of veterans and active-duty service members.

## Prevalence of PTSD and AUD in Military and Veteran Populations

Examined separately, prevalences of PTSD and AUD are high in military and veteran populations when compared with the civilian population. Reports estimate current PTSD prevalence at 6% of predeployed and 13% of postdeployed service members, and from 5% to 13% among veterans, compared to 5% of civilians.^2^–^8^ Lifetime prevalence of PTSD ranges from 7% to 8% among veterans, compared with 6% of civilians.^2^,^8^,^9^ With regard to high-risk drinking, a 2011 U.S. Department of Defense (DOD) survey found that 33% of service members, compared with 27% of civilians, endorsed past-month binge drinking.^10^ Among Operation Enduring Freedom (OEF) and Operation Iraqi Freedom (OIF) veterans, 10% had an AUD diagnosis in their U.S. Department of Veterans Affairs (VA) electronic medical records.^11^

PTSD and AUD often co-occur in military and veteran populations,^2^ as they do in the general population,^12^ and having PTSD or AUD increases the likelihood of experiencing the other.^1^ In national studies, 55% to 68% of veterans with probable PTSD, compared with 40% to 55% of veterans without PTSD, showed evidence of having AUD as well.^2^,^9^ Similarly, among service members and veterans who misuse alcohol, prevalence of PTSD is high. A review of VA electronic medical records indicated that 63% of veterans with AUD and 76% of veterans with comorbid AUD and drug use disorder also had a PTSD diagnosis.^11^

In the general civilian population^13^ and in military and veteran populations, there is evidence that PTSD and AUD are functionally related. For example, in a sample of Vietnam veterans, increases in alcohol use corresponded to increases in PTSD symptom severity,^14^ and veterans with PTSD and substance use disorder (SUD) reported that they perceived that the conditions were interrelated.^15^ Longitudinal studies of veterans have supported the self-medication hypothesis,^16^ which may explain why veterans with unresolved PTSD are more likely to relapse after treatment for substance misuse.^17^

## Factors That Contribute to PTSD and AUD

Among military and veteran populations, the risk for both PTSD and alcohol misuse may vary because of differences in demographic factors, aspects of military culture, and trauma or stress exposure. Relatively little research has addressed risk factors for co-occurring PTSD and AUD. Therefore, we do not know the extent that risk factors may increase the risk for one disorder or both, or whether these risk factors may have additive or interactive effects.

### Demographics

Gender is associated with differential risks for PTSD and AUD. Consistent with the literature on civilians, studies of veteran populations show that lifetime prevalence of PTSD is higher among female veterans (13% to 19%) than male veterans (6% to 7%).^2^,^9^ Civilian men have a higher risk for alcohol misuse than women,^18^ and men are overrepresented in military and veteran populations. Also, male service members report more past-month binge drinking than female service members.^7^,^10^ Despite these gender differences, research on the experiences of women veterans and active-duty service members is limited, and more work is needed in this area.

Racial differences in the prevalence of PTSD have been identified, with higher prevalence occurring among non-White veterans and service members.^2^ In a nationally representative sample of veterans, the lifetime prevalence of PTSD was significantly higher for Black (11%) and Native American veterans (24%), compared with the prevalence for White veterans (6%).^9^ Across military branches, the percentage of service members who reported past-year heavy drinking was similar across Hispanic (9%), White (9%), and African American (8%) groups.^10^

Younger age is associated with higher prevalence of PTSD^9^ and with alcohol misuse.^10^,^16^ For example, a 2011 DOD survey found that among service members ages 18 to 25, 20% endorsed past-year heavy drinking, and 67% endorsed past-month binge drinking.^10^ During a 12-month period, more than 20% of junior enlisted service members experienced serious consequences from alcohol use, including military punishment and arrest.^19^ In a national sample, veterans ages 18 to 29 had the highest odds of a PTSD diagnosis in their lifetimes, and veterans age 65 or older had the lowest odds.^9^ Therefore, the high prevalence of comorbid PTSD and AUD in the military may be due, in part, to the overrepresentation of younger adults in this population.

### Military culture

The military as a whole and each of the military branches have their own distinct cultures, which may influence alcohol-related behaviors and ways to cope with post-traumatic stress. Drinking alcohol is part of military culture as a means for group bonding, recreation, and stress relief.^19^ The drinking behavior of service members and veterans may be influenced by their perception of alcohol consumption norms. For example, in a study among service members who had SUD, the participants tended to overestimate both the average number of drinks consumed by service members and the percentage of service members who were heavy drinkers.^20^

### Military trauma and stress exposure

Researchers have found that military service members and veterans are more likely than civilians to have been exposed to childhood traumatic events, such as physical and sexual abuse and sexual assault, which leads to the suggestion that some individuals enter the military to escape dangerous family environments.^21^,^22^ In particular, one study reported that men with a history of military service had a higher prevalence of exposure to adverse childhood events, especially sexual abuse, than men who had not served in the military.^22^ Childhood stressors also have been associated with high-risk drinking in military recruits,^23^ which may increase vulnerability to stressors encountered during military service.

Veterans and service members report a higher prevalence of trauma exposure than the general population, and they may have a higher likelihood of exposure to specific traumas.^24^ In cross-sectional^25^ and longitudinal studies,^6^ exposure to combat, specifically, has been associated with psychological distress and hazardous drinking. Military sexual assault is also associated with higher PTSD risk than other forms of military and civilian trauma.^26^ According to VA data, about 22% of women and 1% of men report experiencing military sexual trauma, which, in part, may explain the gender differences in the prevalence of PTSD described earlier.^27^

In addition, deployment may expose service members to interpersonal stressors (e.g., separation from social supports and working in close proximity with other service members), mission-related hardship, and prolonged exposure to perceived threats.^25^ Among demobilizing soldiers, 15% reported at least one alcohol-related consequence, and the soldiers’ levels of perceived stress predicted these consequences,^28^ illustrating possible relationships between deployment-related stressors and alcohol misuse.

## Interventions for Prevention of PTSD and AUD

To our knowledge, no study has examined strategies that aim to prevent the development of comorbid PTSD and AUD in military and veteran populations. However, some research has examined the prevention of PTSD or AUD separately in this population, which could inform the prevention of comorbid PTSD and AUD.

### Universal prevention strategies

Universal prevention strategies target all members of a population to prevent the onset of a condition.^29^ According to the *VA/DOD Clinical Practice Guideline for the Management of Posttraumatic Stress Disorder and Acute Stress Disorder*,^30^ no universal prevention strategies for PTSD are currently recommended. Indeed, we know of no research that has tested primary prevention efforts targeting PTSD, AUD, or the comorbid conditions in any population.

### Selective prevention strategies

Selective prevention strategies target members of a population at high risk for developing a condition.^29^ Selective prevention strategies for PTSD involving the use of psychotherapy or pharmacotherapy in the early aftermath of trauma exposure have received some empirical attention, with mixed results.^31^ In general, psychological debriefing interventions have failed to demonstrate beneficial effects in civilian or military samples,^31^,^32^ and in some cases these interventions have been associated with increased PTSD symptom severity.^33^,^34^ In a review of pharmacological selective interventions for PTSD, researchers reported some evidence that hydrocortisone may be effective.^35^ Overall, the VA/DOD practice guideline for PTSD indicates there is insufficient evidence to recommend psychotherapy or pharmacotherapy for selective prevention.^30^ We found no research that has tested selective prevention efforts targeting AUD or comorbid PTSD and AUD in trauma-exposed military populations.

### Indicated prevention strategies

Indicated prevention strategies aim to prevent disorder onset or chronic expression among people already exhibiting symptoms.^29^ Meta-analytic results indicate that trauma-focused psychotherapies involving exposure and/or cognitive restructuring may prevent PTSD among individuals who have acute stress disorder.^31^ However, results are insufficient and mixed regarding the use of pharmacotherapy for the indicated prevention of PTSD.^30^,^36^ For individuals who screen positive for risky alcohol use, providing a single, initial brief intervention about alcohol-related risks and a recommendation to abstain from or moderate drinking may reduce alcohol misuse.^37^,^38^

## Treatment Interventions for PTSD and AUD

Evidence indicates that concurrent treatment of PTSD and AUD can be safe and effective.^30^,^39^ Before reporting on concurrent treatment approaches, we describe evidence-based treatments targeting either PTSD or AUD. We also discuss the efficacy of these treatments for military and veteran populations.

### Treatments for AUD

The *VA/DOD Clinical Practice Guideline for the Management of Substance Use Disorders* recommends using psychotherapy and pharmacotherapy treatments for AUD.^38^ Recommended psychotherapies include cognitive behavioral therapy, behavioral couples therapy, community reinforcement, motivational enhancement therapy, and 12-step facilitation. Recommended pharmacotherapies include acamprosate, disulfiram, naltrexone, and topiramate. Treatment availability and patient preferences are considerations when selecting a treatment.

### Treatments for PTSD

The VA/DOD practice guidelines for treating PTSD recommend using individual, traumafocused psychotherapy.^30^ Pharmacotherapy (i.e., sertraline, paroxetine, fluoxetine, and venlafaxine) and individual psychotherapy that is not trauma-focused are recommended only if trauma-focused psychotherapy is not available or if a patient has a preference. Recommended psychotherapies include prolonged exposure therapy, cognitive processing therapy, and eye movement desensitization and reprocessing. In a recent systematic review of randomized controlled trials, researchers examined the effectiveness of psychotherapy among individuals who had military-related PTSD.^40^ The researchers reported that cognitive processing and prolonged exposure therapies produced large within-group effect sizes, and patients achieved meaningful symptom change, although dropout rates were a problem.

### Concurrent treatments

Veterans with comorbid PTSD and SUD report a preference for integrated treatments that address both conditions simultaneously, and several protocols have been developed to accomplish this.^15^ We found no randomized controlled trials of concurrent treatments for PTSD and AUD conducted in military and veteran populations, but several case studies and small, open or uncontrolled trials provide some preliminary information regarding concurrent treatment in these populations.

#### Psychotherapy

“Seeking safety,” a cognitive behavioral psychotherapy, targets co-occurring PTSD and SUD but is not trauma-focused. Trials of this intervention have had small sample sizes, but the participants, including service members and male veterans, have demonstrated reductions in PTSD symptoms and alcohol misuse.^41^,^42^ One test of this treatment was conducted with female veterans who were homeless.^43^ The participants were not randomly assigned to study conditions, which makes it difficult to determine whether the results were attributable to participant characteristics or treatment effect. When compared with women in the treatment-as-usual condition, women who received the treatment had a greater reduction in PTSD symptoms, but there were no group differences in alcohol use. However, a randomized controlled trial indicated no added benefit of this treatment among male veterans with comorbid PTSD and AUD.^44^ Given that few tests of this treatment have used randomized controlled trials, and findings from other types of studies are mixed, the seeking safety method is not currently recommended for treatment of comorbid PTSD and AUD.^1^,^30^

In one case study of an OEF/OIF veteran, researchers examined the effectiveness of concurrent treatment of PTSD and SUD using prolonged exposure (COPE) therapy.^45^ COPE involves 12, 90-minute sessions that integrate relapse prevention with prolonged exposure therapy. The veteran who received the therapy reported reduced alcohol use throughout treatment, scored in the nonclinical range for PTSD at the end of treatment, and maintained treatment gains at a 3-month follow-up.

Cognitive processing therapy has begun to be examined as a potential treatment for co-occurring PTSD and AUD. This therapy is a 12-session, predominantly cognitive, intervention developed for treatment of PTSD. In a case study, a veteran diagnosed with both PTSD and AUD received cognitive processing therapy that was enhanced to address alcohol use.^46^ The veteran demonstrated clinically significant improvements in PTSD symptoms and alcohol-related problems at the end of treatment and maintained the improvements 12 weeks after treatment. In addition, a review of VA medical records of individuals who received cognitive processing therapy showed no differences for veterans with or without AUD diagnoses in the likelihood of dropping out of treatment, self-reported depression symptoms, or clinician-rated PTSD symptom severity.^47^

Interventions for couples show promise for treating co-occurring PTSD and AUD. Couple treatment for AUD and PTSD (CTAP) is a 15-session manualguided (also known as “manualized”) therapy that integrates behavioral couples therapy for AUD with cognitive behavioral conjoint therapy for PTSD.^48^ In an uncontrolled trial, 13 male veterans and their female partners enrolled, and 9 couples completed the CTAP program. Eight of the veterans showed clinically reliable reductions in PTSD outcomes after treatment. Most of the veterans showed clinically reliable reductions in their percentage of days of heavy drinking.

A couples therapy called “project VALOR,” which stands for “veterans and loved ones readjusting,” involves 25 sessions of cognitive behavioral therapy for PTSD and alcohol misuse, enhanced for significant others. Two OEF/OIF veterans received VALOR therapy in two separate case studies.^49^ These veterans greatly reduced their alcohol use at the start of treatment or shortly before beginning the treatment, and their PTSD symptoms substantially decreased over the course of treatment.

#### Pharmacotherapy

Overall, research on the use of pharmacotherapies for comorbid PTSD and AUD in military and veteran populations is insufficient, and the results are mixed.^30^ For example, in a randomized controlled trial of 30 veterans with comorbid PTSD and AUD, treatment with topiramate, when compared with placebo, was not effective at reducing PTSD symptoms, but the treatment was associated with reduced drinking days.^50^ Also, results from this study indicated that topiramate, when compared with placebo, had a trend-level effect for a reduction in hyperarousal symptoms.

In a double-blind, randomized controlled pilot trial of 9 veterans and 21 civilians, all with comorbid PTSD and AUD, prazosin (which is often used to treat PTSD-related sleep disturbances) did not effectively improve PTSD symptoms.^51^ However, it did reduce the percentage of drinking days. In another double-blind, randomized clinical trial, 96 veterans with comorbid PTSD and AUD received either prazosin or placebo.^52^ In this study, prazosin was not effective in treating PTSD symptoms or reducing alcohol consumption. Overall, prazosin was not effective in treating PTSD symptoms, and its effectiveness regarding alcohol use is unclear. It is possible that alcohol’s effect on sleep interferes with prazosin’s benefits.^51^,^52^

In a double-blind, randomized trial, 88 male veterans with comorbid PTSD and AUD received either paroxetine and naltrexone, paroxetine and a placebo, desipramine and naltrexone, or desipramine and a placebo.^53^ Desipramine outperformed paroxetine in reducing drinking days, and both medications showed some benefit in reducing drinking and core PTSD symptoms, but the addition of naltrexone had no effect on outcomes.

A recent pilot study of *N*-acetylcysteine among veterans with co-occurring PTSD and SUD indicated that *N*-acetylcysteine was associated with significant reductions in both PTSD symptoms and substance craving.^54^ Veterans in this trial received concurrent cognitive behavioral therapy, providing initial evidence for the potential benefit of *N*-acetylcysteine as an adjunct to psychotherapy.

#### Combined psychotherapy and pharmacotherapy

A combination of psychotherapy and pharmacotherapy may be an effective treatment strategy for service members and veterans with comorbid PTSD and AUD. In a single-blind, randomized clinical trial of civilians and veterans with comorbid PTSD and AUD, participants were randomly assigned to receive prolonged exposure therapy and naltrexone, prolonged exposure and a placebo, supportive counseling and naltrexone, or supportive counseling and a placebo.^55^ Participants in all conditions reported reductions in drinking days and PTSD symptoms, and those who received naltrexone had a lower percentage of drinking days than those who received a placebo. There was no statistically significant main effect for prolonged exposure therapy on PTSD symptoms and no observed differences in the number of dropouts across conditions. In the same sample, prolonged exposure was more beneficial for those with non–combat-related traumas and higher baseline PTSD severity.^39^ Also, naltrexone was most beneficial for those with the longest duration of AUD.

## Future Directions for Research and Practice

In research and practice, several notable gaps exist in addressing co-occurring PTSD and AUD in military and veteran populations. First, although military service appears to increase risk for the comorbid conditions, more research is needed to identify factors that contribute to the increased risk for the development of these disorders within the specific military context. In addition, military-specific barriers to accessing care need to be identified. For example, policies that have potential career consequences, such as requiring that treatment participation be recorded in a service member’s military record, may inhibit voluntary participation in treatment. Also, there may be opportunities for prevention during predeployment and postdeployment periods, but research on such programs is scarce. More information about military-specific factors and barriers will help guide prevention and intervention efforts.

Second, although treatments for PTSD and SUD have been disseminated systemwide within the VA, there is a dearth of literature about the effectiveness of these treatments for those in this population who have both conditions. (See Table 1 for brief summaries of treatments that have preliminary reports.) Addressing whether cognitive processing therapy and prolonged exposure therapy can be used for those who have co-occurring PTSD and AUD is a high priority, as existing implementation efforts could be leveraged to address the needs of those with comorbidity.

Comparative efficacy studies also are lacking. Future research should explore which treatments work best for whom, and if matching treatment to patient characteristics improves outcomes. Research on personalized treatment could lead to the development of a menu of evidence-based treatments from which practitioners and patients could jointly tailor a treatment plan for the patient. This menu of treatments could be based on biomarkers, demographics, and other patient characteristics, and it could identify promising alternatives if first-line treatments fail.

Third, it is unclear whether SUD treatments help those who have PTSD. Implementing SUD treatments for individuals with co-occurring PTSD and AUD could be a way for providers to address clinical needs without learning another manual-guided treatment. Motivational enhancement therapy could be used for this purpose, as it has been used successfully to reduce drinking among soldiers with untreated AUD, most of whom also had severe symptoms of PTSD.^56^ This therapy may be useful as an intervention for increasing treatment engagement and preventing treatment dropout. Motivational enhancement therapy also shows promise as a way to increase treatment initiation among veterans and military personnel who are reluctant to enter treatment or address their substance misuse during treatment for PTSD, particularly if they perceive that substance use eases their PTSD symptoms.

Finally, more clinical trials are needed on the treatment and prevention of comorbid PTSD and AUD within military and veteran populations.^57^ Several barriers interfere with the progress of this literature, including the exclusion of people with dual diagnoses, and difficulties recruiting and retaining participants.^50^ Dropout rates for trials testing combined PTSD and AUD treatments tend to be higher than dropout rates for treatment of either disorder alone. Research on the factors leading to participant dropout and on ways of increasing treatment engagement and retention is critical.

## Conclusion

Military and veteran populations have a critical need for interventions that aim to reduce the burden of co-occurring PTSD and AUD. Treating these conditions simultaneously has been challenging and complex in the general population, and military service adds additional risk factors for the likelihood of their onset and maintenance. Although promising interventions exist, more research is needed to assess the degree to which current interventions are effective for service members and veterans. Also, new interventions that target this population should be developed and tested.

## Figures and Tables

**Table 1:** Review of Literature on Treatments for Co-Occurring PTSD and AUD in U.S. Military and Veteran Populations

Treatment	Research Findings
**Pharmacotherapies**	
Desipramine	Reduced drinking and PTSD symptoms in randomized controlled trials.^53^
*N*-acetylcysteine	Observed PTSD symptom reductions in pilot study, as adjunct to psychotherapy.^54^
Paroxetine	Reduced drinking and PTSD symptoms in randomized controlled trials.^53^
Prazosin	Reduced drinking but not PTSD symptoms in pilot randomized controlled trial.^51^ No effects in large randomized controlled trial.^52^
Topiramate	Reduced drinking but not PTSD symptoms in randomized controlled trial.^50^
**Psychotherapies**	
Cognitive Processing Therapy Enhanced for Alcohol Use	Reported symptom reductions in case study.^46^
Concurrent Treatment of PTSD and Substance Use Disorders Using Prolonged Exposure (COPE)	Reported symptom reductions in case study.^47^
Couple Treatment for AUD and PTSD (CTAP)	Observed symptom reductions in uncontrolled trial.^48^
Project Veterans and Loved Ones Readjusting (VALOR)	Observed symptom reductions in case studies.^49^
Seeking Safety	Observed symptom reductions in small trials^41^,^42^ and pre-post trial.^43^ No added benefit in randomized controlled trial.^44^
